# The role and indications of aggressive locoregional therapy in metastatic inflammatory breast cancer

**DOI:** 10.1038/srep25874

**Published:** 2016-05-13

**Authors:** Yi Yan, Lili Tang, Wei Tong, Jingyu Zhou

**Affiliations:** 1 Department of Surgery, The Third People’s Hospital of Chongqing, Chongqing 400014, China; 2 Department of Surgery, Chongqing General Hospital, Chongqing 400014, China; 3 Department of Breast Surgery, Xiangya Hospital, Central South University, Changsha 410008, Hunan, China; 4 Department of Geriatrics Surgery, The Second Xiangya Hospital, Central South University, Changsha 410011, Hunan, China

## Abstract

We seek to confirm the effect and explore the indications of aggressive locoregional management in patients with metastatic inflammatory breast cancer (IBC). Between 2003 and 2014, we reviewed the records of 156 patients with metastatic IBC from five large centers of Breast Surgery in the region of central south of China. Clinicopathologic data were collected to access overall survival (OS), prognostic factors and the indications for locoregional treatment. 75 (48%) patients underwent aggressive locoregional therapy. Patients in locoregional therapy group had a median OS of 24 months compared with 17 months of those in no locoregional therapy group. 2-year OS rate of these two groups was 52% and 32%, separately. Locoregional therapy (HR = 0.556; 95% CI 0.385–0.803; p = 0.002) was confirmed to be an independent prognostic factor, which could significantly improve OS of patients with metastatic IBC. For locoregional therapy group, statistical differences were observed in all subgroups stratified by the factors that were significant in univariate analysis except in the subgroups of stable disease, Charlson comorbidity index ≥3 and cerebral metastasis. Therefore, systemic therapy efficacy, Charlson comorbidity index and cerebral metastasis status appeared to be important indexes for choice of locoregional therapy in different individuals.

Breast cancer has become the leading cancer among women worldwide, which accounts for 25% of female cancer^1^. Due to advanced diagnostic technology and effective multimodality therapy, long term survival of early breast cancer shows great improvement. According to the data from National Cancer Center of China in 2015, the estimated 5-year prevalence for women breast cancer was 1.02 million^2^. Based on this, our research focus has gradually turned to refractory breast cancer.

Inflammatory breast cancer (IBC) is the most fatal form of breast cancer, which accounts for only 1–6% of all breast tumors but roughly 10% of breast cancer motality annually, with an elevated incidence^3^,^4^,^5^. Lacking of specific histological and molecular subtype, the diagnosis of IBC relies mainly on clinical features as follows^6^: rapid and progressive onset of breast erythema, edema or peau d’orange occupying at least one third of the breast (with or without an underlying mass); maximum sympotomatic duration of 6 months; pathologic confirmation of invasive carcinoma.

Due to its aggressive nature, the prognosis of IBC is extremely poor. Multiple recent studies have reported 24–50% metastasis rate and 34–64% 5-year overall survival (OS) rate in IBC patients^7^,^8^,^9^,^10^,^11^. For metastatic IBC, 2-year OS and 5-year OS is 39% and 29–33% respectively^7^,^12^,^13^. Since several important agents such as paclitaxel, docetaxel and trastuzumab were approved for the therapy of breast cancer (BC), and trimodality treatment (anthracycline-based neoadjuvant chemotherapy, modified radical mastectomy, postmastectomy radiation therapy) has gained wide acceptence, the survival of BC patients has obtained great improvement. IBC also benefit from these advances^14^.

For common metastatic BC, aggressive locoregional management were proved to be effective in some retrospective non-randomized clinical studies^15^,^16^,^17^,^18^, such as complete excision of primary breast tumor and radiation therapy to the chest wall and draining lymphatics. Based on these findings, several prospective randomized trials are ongoing. With regard to metastatic IBC, studies are still fairly limited because of less proportion of patients. A recent study confirmed effectivity of primary tumor resection in metastatic IBC^13^.

For this study, we not only analyzed the role of locoregional therapy in metastatic IBC, but also explored the indications for selecting patients who could benefit from this approach. The factors influencing prognosis of metastatic IBC were also researched.

## Methods

### Data Source

Between 2003–2014, we collected 156 metastatic IBC patients’ clinicopathologic data in the region of central south of China from five large centers of Breast Surgery (taxane and trastuzumab were widely used for breast cancer in this developing country since 2003). Multimodality treatment regimens were based on chemotherapy including anthracycline and taxane. Trastuzumab and endocrinotherapy were used in part of patients in line with the indication.

### Ethics approval

The research was reviewed and approved by the Ethics Committee of the Xiangya Hospital of Central South University and was conducted from *June 2003 to June 2014*. This is to certificate that the research design and methods are in accordance with the requirements of regulations and procedures regarding to human subject protection laws such as GCP and ICH-GCP. This study is a retrospective study without any type of clinical intervention.

### Diagnostic criteria

The diagnostic criteria of IBC include sudden onset of inflammation expression of over 1/3 of the range of unilateral breast skin, less than 6 months of duration, and pathologically confirmed as invasive breast cancer^6^. The AJCC Cancer Staging Manual was used to identify TNM-staging^19^. Patients were included only if metastases were found less than 3 months from the diagnosis of IBC.

### Clinicopathological features assessment

Patient’s clinicopathologic features were collected from records, including age, histological classification, menstrual status, Charlson comorbidity index (graded as ≤2 and ≥3), lymph node involvement, status of estrogen receptor (ER)/progestrone receptor (PgR), status of human epidermal growth factor receptor 2 (HER-2), number and site of metastasis, therapeutic regimens, evaluation of clinical response to systemic therapy (graded as complete remission; partial remission; stable disease). Lymph node involvement and clinical response to systemic therapy were assessed by physical examination and imaging examination. Clinical response of no locoregional therapy group was assessed by the result of the last period of systemic therapy. Surgery in this study was for treatment rather than palliative. The endpoint of this study was IBC-related death or the last follow-up date.

### Statistical Analysis

The Kaplan-Meier method was applied to evaluate survival of patients. We used log-rank test to compare survival curves between groups, and stratification analysis to compare survival benefit of locoregional therapy for different subgroups. The patients’ characteristics and associations between groups were analyzed with the Pearson’s Chi squared test. Prognostic factors with p < 0.10 using univariate analysis were entered into the multivariate analysis model. Cox proportional hazards models were fit to assess relationship of these factors and determine independent prognostic factor. Hazard ratios (HRs) and 95% CIs were also recorded. The α-level of 5% was used to determine statistical significance. All tests were two-sided and all statistical analyses were performed by Statistical Package for Social Sciences version 19.

## Results

### Clinicopathologic features of the whole cohort

Among 156 patients, 13 (8%) cases refused to receive any kind of therapy after their diagnosis because of psychological and financial reasons. The rest 143 patients received tanxane and anthracycline-based chemotherapy. 64 (41%) patients presented HER-2 positive and only 21 (33%) of them accepted the treatment of trastuzumab for financial reasons. 70 (45%) of 156 patients demonstrated hormone receptor positive, 61 (87%) of them received endocrine therapy. For locoregional therapy group, 52 (33%) underwent surgical resection of the primary tumor and axillary lymph node. 11 patients of them underwent metastasectomy in the meantime. The time from diagnosis to surgery ranged from 2–15 months. All of them were given adjuvant/neoadjuvant radiation therapy to chest wall and draining nodal volumes. 23 (15%) patients only received locoregional radiation therapy. Among 75 patients treated with radiation therapy, 37 (49%) were delivered a median of 50 Gy in twice-daily fractions of 2 Gy, 15 (20%) patients were delivered a median of 54 Gy in once-daily fractions of 2 Gy, 23 (31%) patients’ regimens were unknown. There were another 21 patients received locoregional symptomatic therapy. Biological agents (pseudomonas aeruginosa injection) were given to 5 patients and interventional embolization were given to 8 patients. The rest 8 patients underwent other forms of palliative surgery. These patients were excluded from locoregional therapy group. Patient characteristics for locoregional therapy group and no locoregional therapy group were summarized in Table 1.

### The factors may lead to selection bias

Patients who underwent locoregional therapy were more likely to have negative Her-2 receptor (67% vs 52%, P = 0.060) and positive locoregional lymph nodes (91% vs 80%, P = 0.067). They were also more inclined to have single metastasis loci than patients who didn’t receive locoregional therapy (76% vs 63%, P = 0.078). Only 6 (8%) patients with cerebral metastasis underwent locoregional therapy, compared with 18 (22%) patients in no locoregional therapy group (P = 0.014). This trend was reversed in patients with soft tissue metastasis (28% vs 16%, P = 0.071).

### Prognosis for locoregional therapy group and matched group

The median OS of all patients was 21 months. 2-year OS and 3-year OS for the entire cohort was 42% and 29% separately. Patients in locoregional therapy group had a median overall survival of 24 months compared with 17 months of those in no locoregional therapy group (p < 0.001, Log-Rank Test). 2-year OS of two groups was 52% and 32% separately. With the exclusive of patients who didn’t receive Herceptin, 21 of 64 cases accepted the treatment of Herceptin. 11 of 21 patients received locoregional therapy. The median OS of this cohort was 28 months. The median OS are 33 months and 15 months separately for locoregional therapy group and no locoregional therapy group (P = 0.055, Log-Rank Test). According to different regimens, patients were divided into 4 groups (surgery + radiation therapy + systemic therapy; radiation therapy + systemic therapy; systemic therapy alone and no therapy). The median OS of four groups was 26 months, 19 months, 18 months and 13 months (p < 0.001, Log-Rank Test).

### The results in univariate analysis

In univariate analysis, a few factors that were significantly associate with OS were as follows: histological grade, Charlson comorbidity index, hormone receptor, Her-2 receptor, systemic therapy efficacy, osseous metastasis, cerebral metastasis and soft tissue metastasis. Other facors, such as locoregional lymph nodes status, number of metastatic sites, visceral metastasis, didn’t significantly influence the prognosis (Table 2).

### The results in multivariate analysis

**Figure 1:**
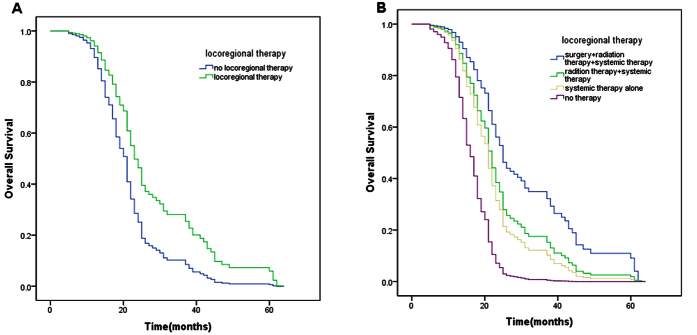
Adjusted other significative factors in univariate analysis. (**A**) Overall survival of patients with metastatic IBC grouped by locoregional therapy (p < 0.01). (**B**) Overall survival of patients with metastatic IBC classified by different regimens (p < 0.01). The two curves were performed in two cox regression models separately.

Prognostic factors with p < 0.10 using univariate analysis entered into the multivariate analysis model. After adjustment of all relevant covariates, locoregional therapy (HR = 0.556; 95%CI 0.385–0.803; p = 0.002) was confirmed to be an independent prognostic factor associated with OS (Fig. 1A). For the purpose of further research in specific locoregional regimens, another multivariable cox regression model was introduced (Table 3). Compared with no therapy group, surgery + radiation therapy + systemic therapy (HR = 0.219; 95%CI 0.090–0.529; p = 0.001), radiation therapy + systemic therapy (HR = 0.362; 95%CI 0.148–0.885; p = 0.026) and systemic therapy alone (HR = 0.438; 95%CI 0.190–1.012; p = 0.053) were associated with a better survival (Fig. 1B). Beside locoregional therapy, other factors such as positive hormone receptor, systemic therapy with CR, osseous metastasis, soft tissue metastasis appeared to be independent prognostic factors for better survival. On the contrary, cerebral metastasis was identified to be associate with a worse survival (Table 3).

### The indications for locoregional therapy in metastatic IBC

**Figure 2:**
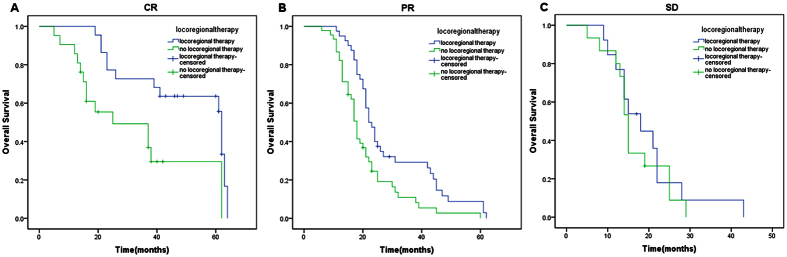
The effect of locoregional therapy in patients with metastatic IBC grouped by according to efficacy of systemic therapy using log-rank test. (**A**) locoregional therapy versus no locoregional therapy in patients with CR (Median OS: 62 months vs 25 months; p = 0.005). (**B**) locoregional therapy versus no locoregional therapy in patients with PR (Median OS: 22 months vs 18 months; p = 0.002). (**C**) locoregional therapy versus no locoregional therapy in patients with SD (Median OS: 18 months vs 15 months; p = 0.534).

**Figure 3:**
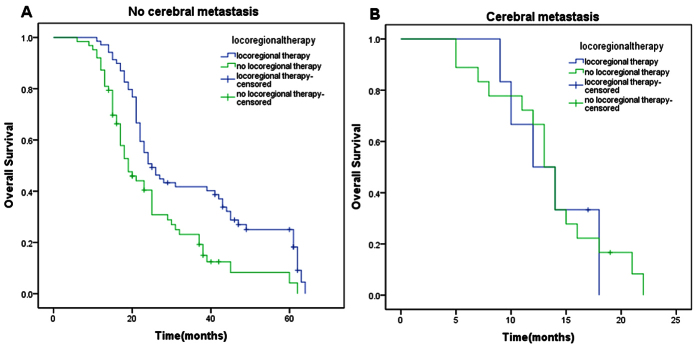
The effect of locoregional therapy in patients with metastatic IBC grouped by cerebral metastasis status using log-rank test. (**A**) locoregional therapy versus no locoregional therapy in patients without cerebral metastasis (Median OS: 25 months vs 19 months; p < 0.001). (**B**) locoregional therapy versus no locoregional therapy in patients with cerebral metastasis (Median OS: 12 months vs 13 months; p = 0.934).

**Figure 4:**
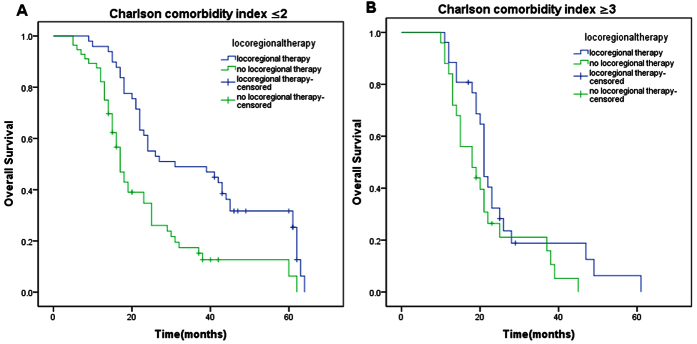
The effect of locoregional therapy in patients with metastatic IBC grouped by Charlson comorbidity index using log-rank test. (**A**) locoregional therapy versus no locoregional therapy in patients with Charlson comorbidity index ≤2 (Median OS: 31 months vs 17 months; p < 0.001). (**B**) locoregional therapy versus no locoregional therapy in patients with Charlson comorbidity index ≥3 (Median OS: 21 months vs 18 months; p = 0.102).

In stratified analysis, the prognostic factors with statistical significance in univariate analyses were divided into subgroups based on different states. For each subgroup, OS was analyzed by Kaplan–Meier method according to locoregional therapy. Finally, we observed statistical differences of OS in patients with CR (Fig. 2A, 62 months vs 25 months, p = 0.005) or PR (Fig. 2B, 22 months vs 18 months, p = 0.002) or without cerebral metastasis (Fig. 3A, 25 months vs 19 months, p ＜ 0.001) between locoregional therapy group and no locoregional therapy group. Patients with SD (Fig. 2C, 18 months vs 15 months, p = 0.534) or with cerebral metastasis (Fig. 3B, 12 months vs 13 months, p = 0.934) didn’t benefit from locoregional therapy. Besides, 2 points of Charlson comorbidity index was defined as a cut-off point. For patients with Charlson comorbidity index ≤2, locoregional therapy could significantly improve OS compared with no locoregional therapy group (Fig. 4A, 31 months vs 17 months, p ＜ 0.001). However, statistical difference wasn’t observed in patients with Charlson comorbidity index ≥3 between these two groups (Fig. 4B, 21 months vs 18 months, p = 0.102).

## Discussion

This retrospective multicentre research analysed the effect of aggressive locoregional management and explore the indications for this approach in metastatic IBC. In order to exclude the impact of different therapeutic regimens in different decades, we collected 156 patients diagnosed after 2003 when drugs like taxane and trastuzumab and multimodality treatment were widely used for breast cancer in this developing country. 75 (48%) patients in this study underwent non-palliative locoregional therapy, while the proportion were 44% and 56% in two related studies recently^10^,^13^. Among these patients, 69%, 58% and 70% of them received both surgery and postmastectomy radiation in our study and these two related analyses separately. This trend is significantly different from earlier studies for common metastatic BC. Lang *et al*.^18^ and Ruiterkamp *et al*.^20^ reported respectively that 24 of 90 (27%) and 256 of 634 (40%) patents with locoregional therapy received both surgery and postmastectomy radiation. With regard to more earlier studies, postmastectomy radiation played a relatively nonsignificant role in common metastatic BC^15^,^21^. They even didn’t mention postmastectomy radiation as a prognostic factor in their studies. But now things have changed, and trimodality treatment is confirmed to improve survival of patients with BC and IBC^6^,^20^. Surgery or radiation therapy alone is increasingly rare, and integrated locoregional regimens are recommended. Therefore, in our research, we made a comprehensive analysis of surgery and postmastectomy radiation rather than surgery alone.

In this study, we found that aggressive locoregional management could improve OS of patients with metastatic IBC in both univariate analysis and multivariate analysis. The results reported 24 months of median OS and 52% of 2-year OS rate in patients with aggressive locoregional management, compared with 17 months and 32% in patients who didn’t receive this approach. Warren *et al*.^10^ revealed in their research a median OS of 2 years for patients with metastatic IBC who received locoregional control, which was similar to our results. Akay *et al*.^13^ reported that primary tumor resection was associated with a nearly 5-fold increase in OS, which showed a stronger effect of locoregional therapy on stage IV IBC. In our analysis, the hazard ration of locoregional therapy group was 0.556 compared to no locoregional therapy group after adjustment of relevant covariates. In contrast with radiation therapy alone, surgical resection of primary tumor combined with postmastectomy radiation therapy were significantly associated with better survival (HR 0.219, 95%CI 0.090–0.529 vs HR 0.362, 95%CI 0.148-0.885). This result was also consistent with the data in Akay’s study mentioned before^13^.

In this analysis, we first explored the indications for locoregional management in metastatic IBC, looking for clinicopathological indexes of patients which suggested benefit from this regimen. Finally, we filtered out three variates as the indexes for selection of locoregional management from significant variates in univariate analysis, which were systemic therapy efficacy, Charlson comorbidity index, and cerebral metastasis status. Systemic therapy could improve OS of patients with metastatic IBC according to our study, which was also proved in other research about stage IV IBC^13^ and BC^21^,^22^. After making a further comparative study, we found that not all the patients with locoregional management could benefit from systemic therapy. Only in patients with CR or PR, locoregional management could bring them better OS. Cerebral metastasis was considerd to have a great impact on prognosis, with the OS ranging from 5 months to 13 months^23^,^24^. In our research, patients with cerebral metastasis couldn’t benefit from locoregional management. We suggest that aggressive locoregional management should be carefully evaluated for these patients. Charlson comorbidity index was first proposed in 1987^25^. The index has gained wide acceptance as a important prognostic parameter. By repeated investigation, we found a cut-off point. When Charlson comorbidity index was no more or more than 2 points, patients could benefit or not benefit from locoregional management. Therefore, for patients with several coexisting illnesses (≥3), we also need to think carefully about the necessity of aggressive locoregional management.

Due to lack of relevant prospective randomized trial, the results of current studies on common metastatic BC were limited by selection bias. Several studies showed that some factors such as primary tumor burden, number of metastatic sites, location of metastasis, response to systemic therapy, performance status and comorbidity index might lead to selection bias^18^,^26^,^27^. Because these factors were often associated with survival, survival benefit of surgery may be a weak argument. A study by Leung *et al*. demonstrated that after adjustment for administration of chemotherapy, no statistical difference was seen between surgery group and no surgery group^28^. Another study also suggested that most of the survival advantage could attribute to selection bias^29^. In our study, we analysed demographic and covariate information of locoregional therapy group and no locoregional therapy group (Table 1). Patients of locoregional therapy group were less likely to have more than one metastasis (P = 0.078), and more likely to have soft tissue metastasis (P = 0.071) and negative Her-2 receptor (P = 0.060). However, these differences didn’t reach statistical significance. Cerebral metastasis between two groups was significantly different (P = 0.014). These factors were analysed in multivariate analysis model. After adjustment of influence by these factors, survival advantage was still seen in locoregional group (Fig. 1A). Because lymph nodes involvement didn’t affect survival in univariate analysis, the variate didn’t entered into multivariate analysis model.

## Conclusion

Aggressive locoregional treatment could significantly improve OS of patients with metastatic IBC. Systemic therapy efficacy, Charlson comorbidity index and cerebral metastasis status appeared to be important indexes for choice of locoregional therapy in different individuals.

## Additional Information

**How to cite this article**: Yan, Y. *et al*. The role and indications of aggressive locoregional therapy in metastatic inflammatory breast cancer. *Sci. Rep.* **6**, 25874; doi: 10.1038/srep25874 (2016).

## Figures and Tables

**Table 1:** Patient demographic information of locoregional therapy group and no locoregional therapy group.

	Locoregional therapy		
	Yes (n = 75)	No (n = 81)	
Variable			P value
Grade			
2	51 (68.0%)	47 (58.0%)	0.198
3	24 (42.0%)	34 (42.0%)	
Charlson comorbidity index			
≤2	49 (65.3%)	56 (69.1%)	0.613
≥3	26 (34.7%)	25 (30.9%)	
Locoregional lymph nodes status			
Positive	68 (90.7%)	65 (80.2%)	0.067
Negative	7 (9.3%)	16 (19.8%)	
Hormone receptor			
Positive	37 (49.3%)	33 (40.7%)	0.281
Negative	38 (50.7%)	48 (59.3%)	
Her-2 receptor			
Positive	25 (33.3%)	39 (48.1%)	0.060
Negative	50 (66.7%)	42 (51.9%)	
Systemic therapy efficacy			
CR	22 (29.3%)	21 (25.9%)	0.891
PR	40(53.4%)	45 (55.6%)	
SD	13 (17.3%)	15 (18.5%)	
Number of metastatic sites			
1	57 (76.0%)	51 (63.0%)	0.078
>1	18 (24.0%)	30 (37.0%)	
Osseous metastasis			
Yes	34 (45.3%)	38 (46.9%)	0.843
No	41 (54.7%)	43 (53.1%)	
Visceral metastasis			
Yes	30 (40.0%)	39 (48.1%)	0.306
No	45 (60.0%)	42 (51.9%)	
Cerebral metastasis			
Yes	6 (8.0%)	18 (22.2%)	0.014
No	69 (92.0%)	63 (77.8%)	
Softtissue metastasis			
Yes	21 (28.0%)	13 (16.0%)	0.071
No	54 (72.0%)	68 (84.0%)	

CR-complete remission, PR-partial remission, SD-stable disease, HER-human epidermal growth factor receptor.

**Table 2:** Prognostic factors of metastatic IBC using a univariate analysis.

Variable	Median overall survival (months)	P value
Grade		
2	23	0.044
3	18	
Charlson comorbidity index		
≤2	23	0.020
≥3	21	
Locoregional Lymph nodes tatus		
Positive	24	0.204
Negative	21	
Hormone receptor		
Positeve	26	<0.001
Negative	18	
Her-2 receptor		
Positive	19	0.021
Negative	23	
Systemic therapy efficacy		
CR	41	<0.001
PR	21	
SD	15	
Number of metastatic sites		
1	21	0.134
>1	21	
Osseous metastasis		
Yes	24	<0.001
No	19	
Visceral metastasis		
Yes	20	0.761
No	22	
Cerebral metastasis		
Yes	13	<0.001
No	23	
Softtissue metastasis		
Yes	28	0.086
No	20	
Locoregional therapy		
Yes	24	<0.001
No	17	

HR- Hazard ratio, CR-complete remission, PR-partial remission, SD-stable disease, HER-human epidermal growth factor receptor.

**Table 3:** Cox proportional hazards models of effect of locoregional therapy.

Variable	HR	95%CI	P value
Grade			
2	1.411	0.866–2.301	0.167
3	1.000		
Charlson comorbidity index			
≤2	1.078	0.701–1.657	0.732
≥3	1.000		
Hormone receptor			
Positeve	1.000		
Negative	2.109	1.375–3.235	0.001
Her-2 receptor			
Positive	1.154	0.766–1.739	0.492
Negative	1.000		
Systemic therapy efficacy			
CR	0.347	0.171–0.702	0.003
PR	0.688	0.405–1.170	0.167
SD	1.000		
Osseous metastasis			
Yes	2.377	1.367–4.134	0.002
No	1.000		
Cerebral metastasis			
Yes	1.000		
No	0.483	0.483	0.029
Softtissue metastasis			
Yes	1.000		
No	3.651	1.924–6.794	<0.001
Locoregional therapy			
Surgery + radiation therapy + systemic therapy	0.219	0.090–0.529	0.001
Radiation therapy + systemic therapy	0.362	0.148–0.885	0.026
Systemic therapy alone	0.438	0.190–1.012	0.053
No therapy	1.000		

HR-Hazard ratio,CI-confidence interval, CR-complete remission,PR-partial remission,SD-stable disease, HER-human epidermal growth factor receptor.
